# Association of Dental College Faculties

**Published:** 1885-09

**Authors:** L. P. Bethel


### ASSOCIATION OF DENTAL COLLEGE FACULTIES.

REPORTED BY L. P. BETHEL, D. D. S.

The second annual meeting of the National Association of Dental Faculties was held at the Sherman House, Chicago, Ill., on Friday and Saturday, July 31st and August 1st.

The colleges represented were as follows :—

Baltimore—R. B. Winder, M. W. Foster.

Pennsylvania Coll. Dental Surgery—C. N. Pierce.

Philadelphia College—S. H. Guilford.

University of Penn., Dent. Dept.—Jas Truman, E. T. Darby.

New York College of Dental Surgery—Frank Abbott.

Boston College of Dental Surgery—J. A. Follett.

Ohio Dental College—H. A. Smith.

University of Michigan—J. Taft, J. A. Watling.

Vanderbilt University—W. H. Morgan.

Iowa University—L. C. Ingersoll, A. O. Hunt.

Kansas City Dental College—J. D. Patterson.

Chicago Dental College—A. W. Harlan, T. W. Brophy.

The meeting was called to order at 11 A. M., Friday, by the President, Dr. C. N. Pierce.

The minutes of the previous meeting were read by the Secretary, Dr. H. A. Smith, and approved.

Roll call was next in order, after which the Secretary read letters from the University of Maryland, acknowledging the receipt of an invitation to unite with the Association. The faculty sanctioned the work of the Association, yet deemed it inexpedient to join, as they believed their instruction was superior to that of other colleges, etc.

On motion of Dr. Truman, the letter was referred to the Executive Committee for investigation. The next letter was from St. Louis Dental College Faculty, Drs. Fuller and Eames stating that they would subscribe to any rule or regulation the Association might adopt, etc.

On motion of Dr. Winder, the Executive Committee was instructed to furnish an order of business, to facilitate the work.

Dr. Abbott presented the following subjects for consideration by the Association, viz :

Preliminary Examinations.

Foreigners entering College.

Admission to the Senior Class.

Question of Fees.

Final Examinations.

Post Graduates.

The subject of Text-books was brought up, and, upon motion of Dr. Abbott, a committee of three was appointed to take the subject into consideration, and to designate them. President Pierce appointed Drs. Abbott, Winder, and Truman to take charge of the matter. It was then moved by J. Taft, that the meeting adjourn to meet at 3 P. M., to hear reports of committees. Carried.

AFTERNOON SESSION—FRIDAY.

The meeting was again called to order at 3 P. M. The minutes of the morning session were read and approved.

The Secretary read a letter from the Illinois Dental College, of Chicago, stating that they had just received a charter, but had organized no faculty as yet, and wished to be instructed as to the course of study they should pursue in their institution. By motion, the communication was laid on the table.

A partial report of the committee was presented, and upon motion, the reports were taken up and acted upon by sections.

The subject of "Preliminary Examinations" was brought up, and

Dr. Taft moved the adoption of the report. He thought that the object of preliminary examinations was, not to ascertain whether the student was or could become qualified to make a dentist, but rather, Has he the attainments that will allow him to go on with his preparatory work? The man who has been trained in theoretical work is better qualified to grasp the subjects brought to his attention, and the man who has the best mind is the one who is best qualified to grapple with the scientific part of the profession. He who has not mind-training and education must learn names mechanically. Those men who are ignorant should not be permitted to enter the profession, or we shall eventually find ourselves without any profession at all.

*Dr. Ingersoll*—I heartily support Dr. Taft's statements. If we do not start men higher up, how can we expect to graduate them higher up? What we want is men with disciplined minds, who are qualified to enter the dental profession.

*Dr. Patterson*—Favored the idea of the Faculties making out a set of questions each year for the different colleges.

*Dr. Foster*—Was not in favor of this suggestion.

*Dr. Taft*—Moved that the action taken last year be referred to a special committee, who shall take the matter in charge, and suggest the particular branches and the feasibility of getting up questions for the colleges, and report at the morning session. An amendment was offered that Drs. Taft, Foster and Ingersoll act as such committee. Carried as amended.

The regular order of business was suspended, and applications for membership were taken up.

Dr. Taft proposed Vanderbilt University, of Tennessee.

Dr. Morgan, of Vanderbilt University, said that owing to circumstances now existing, they could not enter the Association before next year, but they would then be ready to abide by its rulings, and he hoped the Association would admit them at that time.

Referred to a special committee for investigation, on which they reported as follows:

*Resolved*, That in accordance with the statements of Prof. W. H. Morgan, of Vanderbilt University Dental Department, this Association extends to him an invitation to participate in its deliberations, and it will most heartily welcome the school which he represents as a member of this Association, whenever in its judgment it shall apply for such admission.

The Chairman of the Executive Committee, Dr. Frank Abbott, stated that application for admission as a member of this Association has been made by the Vanderbilt University Dental Department, but under the circumstances the committee had concluded not to act upon it until the next meeting, in which decision Prof. Morgan fully concurred.

The regular business was again taken up, and the following resolution was read and adopted:

*Resolved*, That applicants for admission to our senior classes from foreign countries shall be required to furnish, properly attested, evidence of study, of attendance on lectures, etc., the same as is required of the junior students, and to pass the intermediate examinations.

Adjourned.

SATURDAY MORNING—SECOND DAY.

The meeting was called to order at 9.40. The minutes of the previous session were read and approved.

It was recommended that the fees of all colleges, as far as practicable, be made uniform. Recommendation adopted.

It was then recommended that a committee of three be appointed to settle questions that may arise between sessions. Adopted.

It was recommended that three standing committees be appointed, viz: One Executive, one on Curriculum, and one on Text-books. Adopted.

The President appointed the committees as follows: Executive Committee—Abbott, Truman and Taft. Committee on Curriculum—Taft, Brophy and Ingersoll. Committee on Text-Books—Winder, Hunt and Guilford.

A recess of one hour was taken to allow time for the committees to prepare their reports.

Re-assembled at 11.30.

The Committee on Curriculum reported on Preliminary Examinations. Report adopted, as follows:

*Resolved*, That a preliminary examination be required for entrance to our dental colleges; such requirements shall include a good English education. In case of any applicant failing to pass a satisfactory preliminary examination, the other colleges of this Association may be informed of the fact.

*Resolved*, That a candidate for matriculation who presents a diploma from a reputable literary institution, or other evidence of literary qualification, shall be admitted without further examination.

*Resolved,* That for preliminary examinations we recommend the following questions:

1.—Write your name in full.

2.—Give date of birth? Day, Month, Year.

3.—Where were you born? Town, County, State.

4.—Where do you now reside? Town, County, State.

5.—What educational advantages have you had? Name the schools you have attended, and the time spent in each?

6.—What branches have you studied, and to what extent have you pursued them?

7.—In what occupation have you been engaged other than that of student, and how long were you thus employed?

8.—When did you commence studying dentistry? Month, year.

9.—How many months of actual medical and dental study have you had to date?

10.—Have you attended a full course at any medical or dental school? If so, where and when?

11.—With what preceptor, or preceptors, have you studied? Give name and present residence.

Sign your name in full.

Write an English composition, of at least two hundred words, upon a subject of the examiner's selection.

Further examination to be left to the judgment of the different faculties. The examination proposed is to embrace the following:

(1.) English Grammar. (2.) Arithmetic. (3.) Geography. (4.) Modern History. (5.) Government Topics.

A failure to pass the examinations may be communicated to other colleges.

Dr. Taft moved that a committee of three be appointed to draw up a schedule of the different branches and questions, and forward it to the different colleges. Carried.

Drs. Taft, Ingersoll and Harlan were appointed as such committee.

In regard to admission of students into the senior class, the following resolutions were adopted:

*Resolved,* That the Colleges of this Association will receive into the senior class only such juniors as hold certificates of having passed a satisfactory examination in the studies of the junior year, this certificate to be a pledge to any college to which they may apply that a previous term has been properly spent in the institution whence they came.

*Resolved,* That the certificate shall read as follows:

This is to certify that............has attended one full course at the................College of Dental Surgery.............. He was absent from lectures........weeks. He was absent from practical work........weeks. He was examined at the close of the session, and found qualified to enter the senior class.

*Resolved,* That each student upon completing one regular course in any college represented in this Association shall be furnished with the above certificate, without presentation of which he shall not be accepted by any of our colleges for admission to the senior class.

Dr. Wilson then offered the following:

*Resolved,* That a committee of three be appointed to take into consideration the advisability of having a uniform practice in the furnishing of equipments for clinical work. Carried.

Drs. Watling, Wilson, and Darby were appointed as such committee.

Dr. Taft moved that the motion to meet biennially be reconsidered. Carried.

Dr. Taft then moved that the meeting be held one year hence. Carried.

The committee on Text-books then made a report, which was discussed at some length, after which Dr. Taft moved that a committee composed of one member of the faculty of each college in the Association be appointed to take in charge the whole matter of Text-books, and that they shall consider the matter thoroughly, and submit the report at the next regular meeting. The committee was given discretionary power, so long as they did not bring any pecuniary responsibility upon the Association. Carried.

The committee on Text-books was selected as follows:

Baltimore—Dr. Winder.
New York—Dr. Abbott.
Michigan—Dr. Taft.
Boston—Dr. Follett.
Penn. Univ.—Dr. Truman.
Iowa—Dr. Ingersoll.
Missouri—Dr. Eames.
California—Dr. Dennis.
Kansas City—Dr. Patterson.
Philadelphia—Dr. Guilford.
Ohio—Dr. Smith.
Penn. Coll.—Dr. Pierce.
Chicago—Dr. Harlan.

The Treasurer's report was read and referred to the Auditing Committee, who reported favorably. Report adopted.

Moved by Dr. Abbott that the assessment be made three ($3) dollars from each faculty. Carried.

The following were elected as officers for the ensuing year:

C. N. Pierce—President. R. B. Winder—Vice-President.
H. A. Smith—Secretary. A. W. Harlan—Treasurer.

An Executive Committee, consisting of Drs. Abbott, Truman, and Taft, was appointed for the coming year.

The reports of the proceedings were ordered printed.

There being no further business, the meeting adjourned to meet one year hence.

